# Roles for BLyS family members in meeting the distinct homeostatic demands of innate and adaptive B cells

**DOI:** 10.3389/fimmu.2013.00037

**Published:** 2013-02-25

**Authors:** Vishal J. Sindhava, Jean L. Scholz, Michael P. Cancro

**Affiliations:** Department of Pathology and Laboratory Medicine, Perelman School of Medicine, University of PennsylvaniaPhiladelphia, PA, USA

**Keywords:** BLyS, homeostasis, innate B cells, B-1 cells, B-2 cells

## Abstract

B-1 and B-2 B cell populations have different progenitors, receptor diversity, anatomic location, and functions – suggesting vastly differing requisites for homeostatic regulation. There is evidence that the B lymphocyte stimulator (BLyS) family of cytokines and receptors, key factors in the homeostatic regulation of B-2 B cell subsets, is also a major player in the B-1 compartment. Here we review the development and differentiation of these two primary B cell lineages and their immune functions. We discuss evidence that BLyS or a proliferation-inducing ligand (APRIL) availability in different anatomic sites, coupled with signature BLyS receptor expression patterns on different B cell subsets, may be important for homeostatic regulation of B-1 as well as B-2 populations. Finally, we extend our working model of B cell homeostasis to integrate B-1s.

## INTRODUCTION

Immunologic activities are frequently categorized as either innate or adaptive, based largely upon the nature of activating receptors and subsequent effector activity. Classically, innate immune cells such as macrophages and dendritic cells use germline encoded pattern recognition receptors (PRRs) to sense threats. These PRRs interact with conserved, widely distributed microbial structures dubbed pathogen associated molecular patterns (PAMPs; Gordon, 2002). Thus, innate responses provide immediate but broadly targeted effector functions, reflecting the comparatively limited array of invariable PRRs. In contrast, the T- and B-lymphocytes involved in adaptive responses express diverse repertoires of antigen recognition receptors generated through gene rearrangement and point mutation, yielding more precisely tailored, pathogen-specific immune responses.

These broad classifications, originally stemming from competing theories of immune cognition forwarded by Metchnikoff and Ehrlich, continue to evolve and remain robust conceptual constructs. Nonetheless, mounting evidence challenges such simple binary categorization, suggesting instead that cells of the mammalian immune system use a continuum of evolutionarily conserved versus somatically variant receptors, each with distinct developmental, anatomic, and functional characteristics (Herzenberg, 1989; Hirano et al., 2011; Mold and McCune, 2011). Within the B lymphoid lineage, this view is exemplified by the existence of two major developmental populations, B-1 and B-2. While both use somatically generated immunoglobulin (Ig) antigen recognition receptors, they arise from different progenitors, differ in receptor diversity, occupy distinct anatomic niches, and have divergent functions.

As detailed below, these differences suggest that B-1 cells primarily employ an evolutionarily conserved, anticipatory receptor array, are restricted to particular anatomic sites, and target a limited and relatively constant array of self and microbial epitopes. Reflecting these attributes, appropriate B-1 function can be accommodated through the establishment of a small, fixed pool with self-renewal capacity. In contrast, the B-2 pool is primarily tasked with systemic surveillance for unpredictable microbial pathogen variants, thus requiring a large pool size and the continuous turnover of an extensive potential antigen receptor array. These fundamental distinctions pose vastly different requisites for the homeostatic mechanisms governing pool size and composition.

The last decade has witnessed a growing appreciation for how the B lymphocyte stimulator [BLyS, a.k.a. B cell activating factor of the tumour-necrosis-factor family (BAFF)] family of ligands and receptors governs selection and homeostasis, particularly among B-2s and antibody-forming cells (AFCs) derived from either B-1 or B-2 ancestors. These observations prompt the question of whether this molecular family plays analogous roles in the B-1 lineage, and how the unique homeostatic demands of the B-1 niche are accommodated independently of B-2 and AFC niches. Herein we discuss key features of these two B cell populations relevant to their homeostatic requisites, review current knowledge of how the BLyS family governs homeostasis in the B-2 compartment, and consider potential BLyS family member roles in the B-1 compartment. We focus on murine B cell populations, because of the wealth of data afforded by *in vivo* experimental systems, but include information about human B cells as well.

## DEVELOPMENT AND RECEPTOR DIVERSITY IN B LINEAGE POOLS

The B-1 and B-2 cell populations differ in terms of their developmental kinetics as well as antigen receptor repertoires. Two hypotheses have been proposed for the development of B-1 versus B-2 pools. The “separate” lineage model posits distinct, developmentally restricted B-1 and B-2 progenitors, whereas within the “selection” model the two pools share a common progenitor and diverge following ligand-driven selection (reviewed in Montecino-Rodriguez and Dorshkind, 2006). In mice, B-1 cells are generated from fetal liver precursors, and proportionally predominate during fetal and early neonatal development (Hayakawa et al., 1983; Carsetti et al., 2004; Montecino-Rodriguez and Dorshkind, 2006; Montecino-Rodriguez et al., 2006; Yoshimoto et al., 2011). Once established, B-1 B cells undergo self-renewal in the periphery (Deenen and Kroese, 1993; Kantor and Herzenberg, 1993; Piatelli et al., 2003; Ghosn et al., 2011; Yoshimoto et al., 2011). There is mounting evidence that B-1 cells may continue to be produced in adult bone marrow (BM), but with greatly reduced frequency compared to B-2 cell production (Montecino-Rodriguez and Dorshkind, 2006, 2011; Montecino-Rodriguez et al., 2006; Yoshimoto et al., 2011). This early burst of production, followed by self-renewal and/or an ongoing but low rate of B-1 cell differentiation, yields a steady-state B-1 cell pool of comparatively small magnitude (a few million cells per adult mouse; Hayakawa et al., 1986; Lalor et al., 1989; Hamilton et al., 1994). Most current models for peripheral B-1 maturation involve passage through transitional, intermediate developmental stages followed by differentiation to B-1a and B-1b subsets in serous cavities (reviewed in Montecino-Rodriguez and Dorshkind, 2006, 2012; Casola, 2007). In contrast, B-2 B cells are generated primarily in BM following birth, and continue to be produced through the lifetime of the individual (Kantor and Herzenberg, 1993; Carsetti et al., 2004; Ghosn et al., 2011). Constant B-2 cell production, coupled with a relatively long average half-life, yields numbers that achieve steady-state at 8 weeks of age, eclipsing the B-1 pool in overall magnitude (tens of millions of cells per adult mouse; Hayakawa et al., 1983, 1986; Cancro, 2004a).

Both subsets use recombination activating gene (RAG)-mediated somatic recombination of Ig gene segments for antigen receptor expression (Shinkai et al., 1992; Qin et al., 1999). However, the B-1 lineage differs in two key respects. First, their B cell receptors (BCRs) tend to be skewed toward using the smaller, highly conserved J-proximal VH gene segments, such as the murine VH-11 family (Pennell et al., 1989; Pennell, 1995; Seidl et al., 1997, 1999; Herzenberg et al., 2000). Second, their fetally derived progenitors do not engage in N- or P-nucleotide additions, and thus lack appreciable junctional diversity (Gu et al., 1990; Kantor et al., 1997; Lipsanen et al., 1997). Moreover, because they rarely participate in germinal center (GC) reactions engendered by cognate T cell help, their Ig genes rarely undergo somatic hypermutation and only limited isotype switching (Berland and Wortis, 2002; Alugupalli et al., 2004; Griffin et al., 2011). As a result, the array of B-1 receptors is considerably less diverse and, despite employing somatic recombination *per se* for their assembly, represent an essentially germline encoded series of receptors in the mouse. Paradoxically, the Ig genes of human B-1 cells from cord blood show few somatic mutations, but have similar N additions and complementarity determining region 3 (CDR3) lengths when compared to B-2 cells (Griffin et al., 2011).

In contrast to B-1 cells, developing B-2 cells employ the entire VH gene cluster at apparently stochastic rates and undergo extensive junctional diversification through N- and P-nucleotide addition mechanisms (Kantor et al., 1997). Moreover, once receptor expression is achieved following successful IgH and IgL gene rearrangements, developing B-2 cells undergo stringent counterselection against cells with autoreactive or signaling-defective BCRs (Hardy and Hayakawa, 2001). After exiting the BM and passing through an additional selection checkpoint during the transitional developmental stages, newly formed B-2 cells join the mature, naïve compartments as either follicular (FO) B cells or splenic marginal zone (MZ) B cells. The vast majority of these mature B-2 cells are quiescent and thus, unlike the B-1 pool, turnover among B-2 cells is achieved through replacement by newly formed cells, rather than through self-renewal (Cancro, 2004a; Carsetti et al., 2004).

Clues to understanding the underlying basis for such differences in pool size, replacement rates, and receptor diversity may be found in the distinct roles each pool plays in humoral immune function (Montecino-Rodriguez and Dorshkind, 2006; Suzuki et al., 2010). B-1a cells are responsible for so-called natural antibodies (Abs) – Abs to viral or other pathogen epitopes that are present before infection/immunization, as well as for standing IgM and IgA titers to commensal and opportunistic bacteria (Herzenberg, 2000; Alugupalli and Gerstein, 2005; Baumgarth et al., 2005; Montecino-Rodriguez and Dorshkind, 2006). B-1b cells produce Ab upon exposure to pathogens, and confer some degree of immune memory by continued production of IgM (Alugupalli et al., 2004; Alugupalli and Gerstein, 2005; Haas et al., 2005). Natural Abs may recognize self antigens, and thus play a “housekeeping” role through clearance of senescent cells or cell debris (Montecino-Rodriguez and Dorshkind, 2006). While B-1 cells can present antigen, B-1 responses do not involve cognate T help (Martin et al., 2001; Berland and Wortis, 2002; Alugupalli et al., 2004). B-1 cells constitutively express several molecules that are only inducibly expressed by splenic B-2s. For example, B-1a cells have constitutively activated signal transducer and activator of transcription-3 (STAT3), constitutively phosphorylated extracellular signal-regulated kinase (ERK), and upregulated nuclear factor of activated T-cells (NF-AT) and transgelin 2 (Karras et al., 1997). In addition, B-1 cells may be poised to enter cell cycle (Piatelli et al., 2003). Consistent with observations in mice, human B-1 cells spontaneously secrete IgM and show tonic intracellular signaling (Griffin et al., 2011). These observations may begin to explain why peritoneal B-1 cells can proliferate rapidly, sometimes in the absence of BCR-mediated signaling. B-2 cells, on the other hand, participate in inducible responses that often involve T cell help and further receptor diversification through somatic hypermutation of Ig genes within GCs (Chan and Brink, 2012; Victora and Nussenzweig, 2012). In these structures, successive mutation and selection processes culminate in B cells with greatly improved antigen affinity, and class switch recombination leads to Abs of various isotypes, predominantly IgG (Chan and Brink, 2012; Victora and Nussenzweig, 2012). These cells may further differentiate and join long-lived antibody-secreting cell (ASC) or memory B cell subsets (Victora and Nussenzweig, 2012). B-2 B cells are thus the ultimate “source” of the majority of high-avidity and high-affinity, pathogen-targeted Abs. MZ B cells, similar to B-1 cells, have a restricted VH repertoire, are recruited into early immune responses upon antigen activation, and can rapidly differentiate to ASCs without cognate T cell help (Martin et al., 2001; Martin and Kearney, 2002). MZ B cells may also participate in T cell-dependent (TD) GC responses, undergo somatic hypermutation, and differentiate into memory cells (Song and Cerny, 2003; Phan et al., 2005).

In accord with their barrier and housekeeping roles, B-1 cells tend to be localized in particular anatomic niches, especially coelomic cavities and mucosal interfaces (Kroese et al., 1992), and though they can circulate, they are rare in spleen and lymph nodes (Kroese et al., 1992; Montecino-Rodriguez and Dorshkind, 2006). In contrast, the bulk of B-2 cells (members of the FO subset) form quiescent recirculating pools, and in any given response, use only a small (~1/100,000) proportion of available receptors (Cancro et al., 1978). MZ B cells do not recirculate in mice, but their microanatomical location at the splenic red and white pulp border makes them the first B cells to encounter blood-borne pathogens (Martin et al., 2001; Belperron et al., 2007; Kearney, 2008).

An exhaustive analysis of B-1a, B-1b, and B-2 cells from the murine peritoneal cavity indicates that selective pressures in the periphery, particularly in certain anatomic locales, further shape the repertoires of all of these B cell subsets (Vale et al., 2010). For example, cells of all of these subsets that have undergone N-addition share many similarities in repertoire, including enrichment for adult BM CDR3 sequences. This supports both the hypothesis of different developmental origins as well as a mechanism of BCR-influenced selection as factors that shape B-1 and B-2 subset repertoires, and make the peritoneal cavity – or other anatomic locales – more specialized in terms of the antigens recognized and immune responses undertaken (Montecino-Rodriguez and Dorshkind, 2006, 2012; Vale et al., 2010).

These differences in the generation, repertoire diversification, anatomical location, and immunological function of B-1 and B-2 populations suggest that cells of the B-1 and B-2 lineages have very different homeostatic requisites. The smaller, self-renewing B-1 population, with its relatively limited and static receptor array, is positioned to make sustained and commonly required antibody responses – including to evolutionarily “predictable” microorganisms such as endosymbionts. Continual production of B-2 cells yields a very large and qualitatively diverse B-2 population well-suited to surveillance and antibody responses against novel, evolutionarily unpredictable pathogens. The BLyS family of cytokines and receptors is important for homeostatic regulation of mature naïve and antigen-experienced B-2 subsets, and there is evidence that these same molecules also play a role in B-1 homeostasis. For the remainder of this review, we discuss a working model for how the BLyS family may integrate B cell homeostasis across both primary lineages.

## THE BLyS FAMILY PLAYS KEY ROLES IN B-2 B CELL HOMEOSTASIS AND HUMORAL IMMUNE RESPONSES

**FIGURE 1:**
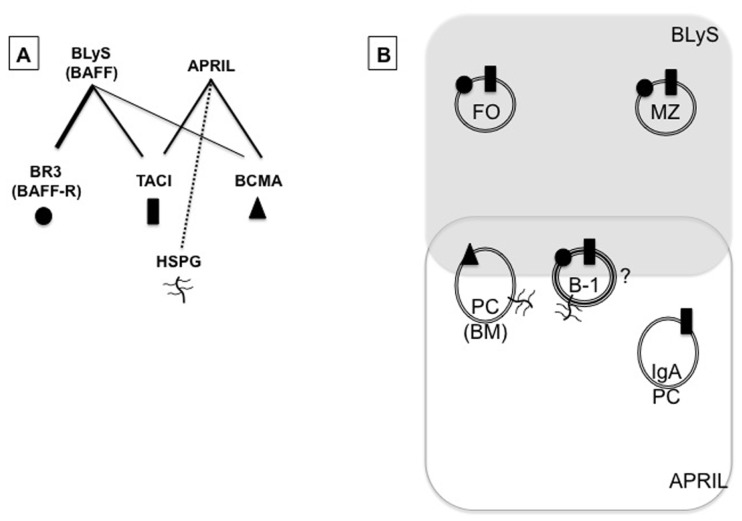
**BLyS family members (except for HSPG) and model for BLyS family roles in B cell homeostasis**. **(A)** BLyS binds to all three receptors – BR3 (circle), TACI (rectangle), and BCMA (triangle) – with relative affinity indicated by line thickness. APRIL binds only to TACI and BCMA, but also to HSPGs expressed on B cells. **(B)** Diagrammatic representation of potential homeostatic niches, governed in part by BLyS or APRIL signaling through specific BLyS receptor(s). Persistence of naïve follicular (FO) and marginal zone (MZ) B cells is governed by BLyS signaling through BR3, yet cells of these subsets also express TACI, and there is evidence that APRIL signaling through TACI is key for isotype switching to IgA following antigen encounter. BCMA is required for long-lived plasma cell (PC) maintenance in bone marrow (BM) likely by APRIL signaling through this receptor, but BLyS may also play a role. B-1 B cell maintenance may require BLyS in the spleen but APRIL in the peritoneal cavity.

The tumor necrosis factor (TNF) superfamily cytokines, BLyS (also known as BAFF) and a proliferation-inducing ligand (APRIL), are key regulatory factors in B cell survival and function (Moore et al., 1999; Schneider et al., 1999; Gross et al., 2001; Schiemann et al., 2001; Castigli et al., 2004; Belnoue et al., 2008). BLyS binds to three receptors: BLyS receptor 3 (BR3; also known as BAFF-R), transmembrane activator-1 and calcium modulator and cyclophilin ligand-interactor (TACI), and B cell maturation antigen (BCMA). In contrast, APRIL binds to TACI and BCMA (Cancro, 2004b; Bossen and Schneider, 2006), and to heparan sulfate proteoglycans (HSPGs; Hendriks et al., 2005; Ingold et al., 2005). These relationships are summarized in **Figure 1A**. Known or potential roles (indicated by a question mark) of various receptor-ligand combinations discussed in this review are outlined in **Table 1**.

**Table 1:** Roles for cytokine-receptor signals in murine B cell homeostasis.

	BLyS	APRIL
BR3	• Transitional selection (Hsu et al., 2002; Crowley et al., 2008; Stohl et al., 2011) • FO, MZ survival (Lentz et al., 1996; Gross et al., 2001; Hsu et al., 2002) • GC evolution (Schiemann et al., 2001; Rahman et al., 2003; Vora et al., 2003) • TLR/BCR crosstalk, regulation? (He et al., 2010; Oropallo et al., 2011) • B-1 maintenance in spleen? (Lentz et al., 1996; Ng et al., 2006; Scholz et al., 2008; Mackay et al., 2010)	
TACI	• Class switch (Litinskiy et al., 2002; Castigli et al., 2005) • B-1 maintenance in spleen? (Lentz et al., 1996; Ng et al., 2006; Scholz et al., 2008; Mackay et al., 2010) • Integration of TLR signals? (Litinskiy et al., 2002; He et al., 2010; Oropallo et al., 2011)	• IgA class switch, B-2 and B-1 cells (Litinskiy et al., 2002; Stein et al., 2002; Castigli et al., 2004; Sakurai et al., 2007; Kataoka et al., 2011) • Short-lived ASC responses (Castigli et al., 2005; Yang et al., 2005) • B-1 maintenance in the peritoneum? (Gross et al., 2001; Shulga-Morskaya et al., 2004) • APRIL-HSPG-TACI signaling? (Sakurai et al., 2007; Kimberley et al., 2009)
BCMA	• Long-lived plasma cell generation and/or maintenance (O’Connor et al., 2004; Ingold et al., 2005; Benson et al., 2008) • Memory B cell recall response? (Avery et al., 2003; Darce et al., 2007a,b; Ettinger et al., 2007; Scholz et al., 2008)	• Long-lived plasma cell homing to or maintenance in BM (O’Connor et al., 2004; Belnoue et al., 2008; Benson et al., 2008)
HSPG		• B cell maturation (Reijmers et al., 2011) • IgA class switch (Sakurai et al., 2007) • Plasma cell survival (Reijmers et al., 2011) • B-1 maintenance in the peritoneum? (Gross et al., 2001; Shulga-Morskaya et al., 2004)

BLyS is the biological metric of space for the pre-immune B-2 cell pool: more BLyS equates with higher absolute numbers of FO and MZ B cells (Moore et al., 1999; Hsu et al., 2002; Stohl et al., 2005). BLyS is expressed by monocytes, macrophages, dendritic cells, and neutrophils, but not by B cells themselves (Moore et al., 1999; Kawasaki et al., 2002; Litinskiy et al., 2002; Gorelik et al., 2003; Scapini et al., 2003). However, the major source of systemic BLyS is likely radiation-resistant stromal cells of secondary lymphoid organs; thus linking BLyS amounts to body volume and consistent with the observation that larger animals have more B cells than smaller ones (Gorelik et al., 2003). BLyS is a 285-amino acid type II transmembrane protein, which is cleaved at a furin consensus site and released as a soluble trimeric ligand (Moore et al., 1999; Schneider et al., 1999). Both the trimer and a 60-mer structure (composed of 20 trimers) are detected in serum and appear to be biologically active. For example, the BLyS trimer binds with highest affinity to BR3, whereas BLyS 60-mers are the more efficient TACI agonist (Bossen et al., 2008).

Unlike BLyS, APRIL has no effect on primary B-2 cell numbers (Castigli et al., 2004; Varfolomeev et al., 2004). This may reflect redundancy with BLyS, and/or roles for APRIL among antigen-experienced B cell subsets, as discussed further below. APRIL is expressed by monocytes, dendritic cells, macrophages, T cells, eosinophils, osteoclasts, and BM stromal cells (Hahne et al., 1998; Mukhopadhyay et al., 1999; Nardelli et al., 2001; Litinskiy et al., 2002; Moreaux et al., 2005; Chu et al., 2011). APRIL is a 250-amino acid type II transmembrane protein processed within the Golgi apparatus by furin convertase and released as a trimeric biologically active form; it does not form soluble 60-mers like BLyS (Lopez-Fraga et al., 2001). However, an intergenic splicing between *tweak* and *april* genes generates TWE-PRIL protein, which may exist as a membrane bound form (Pradet-Balade et al., 2002).

Homeostasis of the mature naïve B-2 compartment is governed by the BLyS–BR3 axis (reviewed in Crowley et al., 2008; **Table 1**). BLyS–BR3 signaling integrates BCR-mediated negative selection at the transitional developmental checkpoint with homeostasis of the mature naïve compartment. BLyS is a limiting survival factor, and only those cells that successfully compete for it persist in the FO or MZ B cell pools. If BLyS levels are reduced or BR3 signaling is abrogated, fewer cells survive the transitional checkpoint, and numbers of FO and MZ B cells are reduced or, in the case of BLyS knockouts, virtually non-existent (Miller and Hayes, 1991; Lentz et al., 1996; Gross et al., 2001; Schiemann et al., 2001; Thompson et al., 2001; Yan et al., 2001). Conversely, if BLyS levels are experimentally elevated, the stringency of transitional selection is “relaxed,” and cells that would normally be selected against – such as those with autoreactive BCRs – instead survive to join the FO or MZ pool (Stohl, 2005; Stohl et al., 2005). Numbers of cells in each of these subsets are correspondingly increased (Hsu et al., 2002). Thus, the BLyS–BR3 axis also plays a key role in peripheral tolerance (Stohl, 2005; Stohl et al., 2005). The number of B cells reaches several million (MZ) or tens of millions (FO) in the adult mouse. These cells are quiescent and reside in the compartment for the duration of their lifespan (measured in months) unless they respond to antigen. Thus, the developing B-2 lineage yields a mature naïve compartment of sufficient steady-state size and diversity for effective immune surveillance, along with sufficient quality to avoid autoreactivity. Mounting evidence indicates that the BLyS–BR3 axis governs primary B cell homeostasis in humans as well: naïve mature human B cells express BR3 and bind BLyS; people with a homozygous BR3 deletion have few mature B cells; and mature naïve B cells are ablated with anti-BLyS therapy (belimumab; Carter et al., 2005; Darce et al., 2007a,b; Palanichamy et al., 2009; Warnatz et al., 2009; Calero et al., 2010).

Members of the BLyS family also play roles in humoral immune responses undertaken by B-2 B cells in both mice and humans. BLyS is essential for humoral immune responses in mice, as blocking BLyS signaling leads to significantly impaired TD and T cell-independent (TI) immune responses (Gross et al., 2001; Schiemann et al., 2001; Vora et al., 2003; Scholz et al., 2008). APRIL is also suggested to have roles in immune responses. For example, APRIL transgenics show significantly elevated antigen-specific IgM and IgG in response to a TI type 2 antigen (Stein et al., 2002), and APRIL plays a key role in IgA class switch and the IgA antibody response to mucosal and TI type 1 antigens (Castigli et al., 2004). Knockout mice show TACI is the BLyS family receptor important for APRIL-mediated IgA class switching (Castigli et al., 2004; Sakurai et al., 2007), though other work shows that the BLyS–TACI interaction also mediates isotype switch (Castigli et al., 2005). *In vitro* studies indicate that APRIL synergizes with BCR cross-linking to induce B cell proliferation, and also induces the upregulation of co-stimulatory molecules and the antigen presentation function of B cells (Yu et al., 2000; Yang et al., 2005). Either BLyS or APRIL (with other appropriate cytokines) elicits TI-like class switching in cultured human B cells (Litinskiy et al., 2002). Interestingly, a range of mutations in TACI is associated with common variable immune deficiency (CVID) and IgA deficiency (Castigli et al., 2007; Lobito et al., 2011; Martinez-Gallo et al., 2013). Furthermore, there is increasing evidence in both mice and humans that TI responses to many innate-like antigens occur through pathways that integrate BLyS or APRIL signaling through TACI with Toll-like receptor (TLR) signaling (He et al., 2010; Oropallo et al., 2011).

Heparan sulfate proteoglycans have been identified as an APRIL-specific binding receptor on both B-1 and B-2 cells (Hendriks et al., 2005; Ingold et al., 2005). APRIL contains a short, basic amino acid sequence that is absent in BLyS; in association with additional basic residues these are required for APRIL binding to negatively charged HSPGs. Crystal structures reveal that the HSPG binding site of APRIL is clearly distinct from that of TACI and BCMA; and APRIL binding to HSPG is not inhibited by BCMA:Fc (Wallweber et al., 2004; Hendriks et al., 2005; Ingold et al., 2005). HSPG-mediated binding of APRIL is essential for B cell proliferation and IgA production in collaboration with TACI (Sakurai et al., 2007), raising the possibility of ternary complex formation of APRIL–TACI–HSPG. In this regard, a study by Kimberley et al. (2009) showed that HSPG serves as a platform for APRIL multimerization on the cell surface, which can then signal through TACI for optimal activation of B cells.

Germinal centers, a hallmark of TD immune responses, generate high-affinity, class-switched antibody as well as persistent (long-lived) plasma cells and memory B cells (Victora and Nussenzweig, 2012). GCs begin in the absence of BLyS or BR3, but are not maintained – likely reflecting the paucity of naïve B cells available for recruitment into the ongoing GC reaction, but also suggesting a role for BLyS–BR3 signaling in GC evolution (Rahman et al., 2003; Vora et al., 2003). Likewise, high-affinity, class-switched antibody responses are significantly reduced, and the memory response is impaired (Schiemann et al., 2001). Studies of APRIL knockouts in different genetic backgrounds report disparate effects upon TD antigen challenge: either a normal humoral response, or enlarged GCs and an enhanced IgG response (Castigli et al., 2004; Varfolomeev et al., 2004). There is increasing evidence that both BLyS and APRIL are required for plasma cell generation and maintenance (Ingold et al., 2005; Benson et al., 2008), and that APRIL is important for plasma cell homing to or retention in the BM (Ingold et al., 2005; Belnoue et al., 2008). Studies of BCMA knockouts show that this receptor is critical for maintenance of long-lived plasma cells in BM (O’Connor et al., 2004). In addition, HSPGs are key for APRIL-mediated plasma cell survival (Reijmers et al., 2011).

Memory B cells appear to occupy a BLyS-independent homeostatic niche in mice. Neither BLyS nor APRIL is required for memory B cell maintenance in mice treated with anti-BLyS, BR3-Ig, or TACI-Ig, though unswitched (IgM) memory cells are less resistant to BLyS neutralization (Benson et al., 2008; Scholz et al., 2008). In contrast, BLyS promotes survival of resting human memory B cells and plasmablasts (Avery et al., 2003; Ettinger et al., 2007; Karnell and Ettinger, 2012). BLyS treatment of human memory B cells (CD27^+^) *in vitro* attenuates ASC formation with TI-like stimulation but enhances ASC formation with TD-like stimulation (Darce et al., 2007a), and activated cells upregulate BCMA (Darce et al., 2007b), again pointing to roles for BLyS family members in human plasma cell differentiation. BCMA and/or TACI expression have been reported on murine and human memory B cells, but elucidation of expression levels and patterns – as well as homeostatic requisites – awaits definitive characterization of memory B cell subsets (Darce et al., 2007b; Benson et al., 2008; Tomayko et al., 2010; Scholz et al., 2011).

## BLyS FAMILY MEMBERS MAY PLAY ROLES IN B-1 B CELL HOMEOSTASIS

The molecules that govern generation, maintenance, and selection of B-1 subsets are under active investigation. However, there is increasing evidence that BLyS family members play analogous but distinct roles in B-1 and B-2 pools. Peritoneal B-1 cells of mice express both BR3 and TACI, but barely detectable surface BCMA (Ng et al., 2006). Nevertheless, BR3 knockouts, BR3 signaling-mutant mice, and BLyS knockouts all have wild-type or moderately (not significantly) reduced numbers of B-1 cells in the peritoneal cavity (Lentz et al., 1996; Schneider et al., 2001; Sasaki et al., 2004; Shulga-Morskaya et al., 2004; Gavin et al., 2005). BLyS-overexpressing mice show a significant increase in splenic B-1 numbers and peritoneal cavity B-1 percentages (Gavin et al., 2005), consistent with some prior reports (Gross et al., 2001), but not others indicating similarity between B-1 populations in BLyS transgenic and wild-type mice (Mackay et al., 1999). Interestingly, mice in which BLyS was neutralized had reduced splenic B-1 numbers but normal peritoneal B-1 numbers (Scholz et al., 2008), suggesting the possibility of different homeostatic requisites in different anatomic environments. There was a significant decrease in peritoneal B-1 numbers in mice transgenic for TACI-Ig, a molecule that neutralizes both BLyS and APRIL (Gross et al., 2001). In contrast, TACI-Fc transgenic mice showed only a negligible decrease in peritoneal B-1 subsets (Schneider et al., 2001). B-1 transitional cells are not dependent on BR3 signaling, although similar to B-2 transitional cells, BCR signaling is required for survival and continued maturation (Mackay et al., 2010; Montecino-Rodriguez and Dorshkind, 2011). BLyS treatment of B-1 cells stimulated with TLR ligands leads to upregulated BR3 and TACI expression, increased proliferation, and cytokine secretion (Ng et al., 2006). Together these results suggest that neither BR3 nor BLyS is absolutely necessary for B-1 cell development, selection, or survival in the periphery, though BLyS may modulate B-1 cell maintenance or responses in certain anatomic sites, for example through localized BLyS production by myeloid cells (Gavin et al., 2005; **Table 1**).

Clearly, peritoneal B-1 cells are far less dependent on BLyS than are B-2 cells. One possible explanation is that peritoneal B-1 cells require alternative or redundant survival factors, for example, APRIL (discussed further below). Unlike splenic B-2 cells, peritoneal B-1 cells are constitutively active with regard to surface and intracellular activation markers and spontaneous IgM secretion (Fischer et al., 2001; Kretschmer et al., 2003; Tumang et al., 2004); and activated B cells may have different survival requisites than quiescent cells. Splenic and peritoneal B-1a cells both express CD5, but only peritoneal B-1 cells respond to PMA as shown by cell cycle progression, Ig secretion, and expression of Notch-related genes (Fischer et al., 2001; Tumang et al., 2004). As noted above, peritoneal but not splenic B-1 cells express a constitutively activated form of STAT3, which regulates IL-10 gene expression (Fischer et al., 2001). In addition, the microenvironment of the peritoneal cavity is thought to be very different from that of the spleen, and may contain redundant or alternative survival factors that can substitute for BLyS. Therefore, it is tempting to speculate that splenic and peritoneal B-1 cells are homeostatically different from each other as well as from B-2 subsets and may require different signal/s for survival.

Likewise, there is evidence, albeit indirect and in some cases conflicting, that APRIL may play roles in B-1 B cell maintenance or responses. Indeed, B-1 B cells express BR3 and TACI (Ng et al., 2006), and in one report, peritoneal B-1 cells expressed a higher level of TACI compared with peritoneal B-2 cells (similar to the higher level of TACI expressed by MZ B cells compared to FO B cells; Shulga-Morskaya et al., 2004; Ng et al., 2006; Stadanlick et al., 2008). Nonetheless, TACI knockout mice have normal numbers of peritoneal B-1 cells (von Bulow et al., 2001). APRIL knockout mice were reported to have normal percentages of peritoneal B-1 cells (Castigli et al., 2004), yet APRIL transgenic mice had increased percentages of splenic B-1 cells (Planelles et al., 2004). APRIL knockout mice also have significantly decreased serum IgA levels as well as IgA responses to orally administered antigen (Castigli et al., 2004); however, although IgA is an isotype normally associated with B-1 cells, it is not known if B-2 as well as B-1 cells were involved in these responses. There are conflicting reports of B-1 cell prevalence in mice that should have been chronically depleted of both BLyS and APRIL using soluble receptor transgenes (Gross et al., 2001; Schneider et al., 2001). The discrepancy in these observations may have been due to differences in the transgenes and expression cassettes employed; or to insufficient levels of soluble receptor for complete neutralization of both BLyS (including BLyS 60-mers) and APRIL. *Ex vivo* observations and cell culture experiments indicate that mucosal dendritic cells upregulate APRIL production, and B-1a cells upregulate TACI and undergo enhanced class switching to IgA in a mucosal, TI immune response (Kataoka et al., 2011), echoing roles for APRIL and TACI in TI responses of B-2 B cells. Taken together, these observations suggest potential roles for APRIL in B-1 cell survival, activation, or function, but comprehensive analyses will be required to clarify the mechanisms involved. There are several approaches that might be taken to definitively establish the role of APRIL in B-1 cell origin/maintenance, such as detailed studies of B-1 cell numbers in APRIL knockout mice, or enumeration of B-1 cells in BCMA:Fc transgenic mice or following BCMA:Fc treatment of wild-type mice. Furthermore, it will be important to examine the role of the APRIL–HSPG axis, since B-1 cells express TACI, and HSPGs can serve as a platform for APRIL multimerization and cross-linking with TACI (Kimberley et al., 2009).

## SUMMARY

The two major B lymphocyte lineages, B-1 and B-2, together provide an array of antigen recognition receptors for “expected” endogenous antigens as well as “unexpected” novel/exogenous antigens; patrolling (circulating) as well as location-specific surveillance and persistence; constitutive as well as inducible Ab production; and rapid as well as longer-term humoral responses. BLyS family molecules play key roles in the maturation and maintenance of B-2 B cell subsets in the periphery, and there is increasing evidence that this same family plays similar roles for cells of the B-1 lineage. Therefore, we now include B-1 cells in our working model for BLyS-mediated homeostatic control, outlined in **Figure 1B** (Crowley et al., 2005, 2008; Miller et al., 2006; Treml et al., 2006, 2009). B-1 cells are placed in the area of overlap between the “BLyS space” and the “APRIL space” based on mounting evidence that BLyS and/or APRIL modulate B-1 cell maintenance or responses in different anatomic locales. For example, a role for APRIL in B-1 survival is suggested by one report of a nearly complete absence of peritoneal B-1 cells in TACI-Ig transgenic mice, compared with the smaller or localized effects on peritoneal B-1 numbers and lifespan in BLyS-deficient, BLyS-neutralized, or BR3 signaling-deficient animals; whereas more nuanced roles are indicated for BLyS and B-1 cell survival or function in the spleen. APRIL is further implicated by the expression of HSPG on peritoneal B-1 cells.

This model for regulation of steady-state numbers of various B cell subsets is based on BLyS or APRIL availability and BLyS receptor expression patterns. For example, ample experimental evidence shows that systemic, trimeric BLyS acting through BR3 regulates selection thresholds and lifespan in the periphery, thus achieving steady-state numbers of mature naïve FO and MZ B cells. Differential expression of BLyS receptors during activation and further differentiation to ASC or memory B cells provides related, yet distinct, homeostatic niches. Localized cytokine sources may afford survival requisites, as has been suggested for the persistence of long-lived plasma cells in BM and for the maintenance or expansion of B-1 cells (Gavin et al., 2005; Chu et al., 2011). Indeed, existing evidence suggests that whereas BLyS is key for naïve B-2 cells, APRIL is important during or following differentiation to ASCs (**Figure 1B**). Additional molecules yet to be identified are likely involved in establishment and maintenance of antigen-experienced B cell subsets, possibly molecules whose expression is restricted to specific anatomic milieus, including long-lived plasma cells, memory B cells, and B-1 cells. Nevertheless, it is becoming increasingly clear that the BLyS family may elegantly integrate homeostasis of both B-1 and B-2 lineages.

## Conflict of Interest Statement

The authors declare that the research was conducted in the absence of any commercial or financial relationships that could be construed as a potential conflict of interest.
